# Exploring engagement, well-being, and welfare on engagement platforms: Insight into the personal service sector from the DACH region

**DOI:** 10.1007/s12525-022-00589-1

**Published:** 2022-09-23

**Authors:** Simon Michalke, Lisa Lohrenz, Christoph Lattemann, Susanne Robra-Bissantz

**Affiliations:** 1 Jacobs University Bremen gGmbh, Management and Economics, Campus Ring 1, Bremen, 28759 Germany; 2 TU Braunschweig, WI2, Mühlenpfordtstraße 23, Braunschweig, 38106 Germany

**Keywords:** Platforms, Service logic, Engagement, Social welfare, Well-being, Engagement platforms, O330

## Abstract

Engagement platforms (EPs) are an essential technology to enable co-creation and service innovation. Therefore, the design and governance of these platforms are receiving increasing attention in research. In this study, we aim to identify which activities and mechanisms foster engagement and which governance mechanisms are implemented to avoid harm on EPs. To this end, we conducted expert interviews with founders, CEOs, and managers of 14 personal and household-related service platform companies from the DACH region (Germany(D), Austria(A), Switzerland(CH)), to gain insights into their activities and mechanisms for creating and maintaining successful EPs. We found eight mechanisms, e.g., moderation of content, limitations of entry and certification, employed by personal EPs (PEPs) as self-regulatory mechanisms to avoid misconduct and negative experiences of actors. The identified governance mechanisms may guide the design and governing of PEPs by providing tangible examples to foster actor engagement while considering externalities on a societal and individual level.

## Introduction

Over the past two decades big tech organizations e.g. Apple, Google, and Microsoft were able to create dominating technological platforms and ecosystems that provide a wide array of highly convenient service experiences to their users and partners (Bazarhanova et al., 2020; Cusumano, 2014; Evans & Gawer, 2016). The quality and convenience of these services, however, rely on the continuous engagement of heterogeneous actors who contribute tangible and intangible resources to their own benefit or that of others (Vargo et al., 2008; Vargo & Lusch, 2004). Therefore, we adopt the conceptualization of engagement platforms (EPs) in this research to assess the role of engagement in the context of digital platforms and ecosystems. Engagement is “a psychological state that occurs by virtue of interactive, co-creative customer experiences with a focal agent/object in a particular service relationship” (Brodie et al., 2011, p. 259). EPs are physical or virtual points of contact that structurally support the value co-creation of loosely coupled actors in dynamic engagement ecosystems (Breidbach et al., 2014).

As a result of digitalization in recent years, many face-to-face interactions are now orchestrated via virtual points of contacts (Breidbach et al., 2014), while traditional sectors, such as health care, still largely rely on physical means of contact, i.e. interpersonal interfaces utilizing hardware and facilities. Thus, the conceptualization of EPs affords the assessment of how individual touchpoints, both physical and virtual, form overarching engagement ecosystems to improve the quality of interactions. E.g., Google implemented EPs, such as android smartphones or stores (physical), as well as, the Google Play Store or Youtube (virtual) (Breidbach et al., 2014). While EPs are employed across all industries and domains, we focus on the personal service context. The personal service context heavily relies on interpersonal interaction related to often personal and household-related settings (Bitner et al., 2000; Lattemann et al., 2019; Woodside et al., 1998). In this context, e.g. a neighborhood community may implement an app to initiate an interaction with other actors as a virtual touchpoint, while community events in physical locations serve as points of contact.

Personal services often inherently rely on the physical interaction with humans or humans with intermediary information systems (Guerrero et al., 2020). Therefore, considering both physical and virtual touchpoints is central to our understanding of EPs as physical (non-technological and technological) and virtual elements allow and foster value co-creation of actors. The concept of EPs also acknowledges several central characteristics of digital platforms (Breidbach & Brodie, 2017), i.e., as extensible codebase (Tiwana, 2015), software and hardware (Tilson et al., 2010), as well as, aspects related to multi-sided platforms that specifically consider value co-creation of distinctive actor groups, such as sellers and buyers (Boudreau & Hagiu, 2009). By specifically considering how engagement is fostered over multiple interactions relying on both virtual and physical touchpoints (Breidbach et al., 2014), the conceptualization of EPs affords a fitting lens to examine platform mechanisms and engagement-related effects (Storbacka, 2019). Employing the concept of EP also excludes digital platforms that mainly enable machine to machine interaction, e.g. Internet of things platforms, but focus on socio-technological use cases.

Given the ongoing debate on the negative effect of EPs on societal and individual welfare (Clemons et al., 2021; Lohrenz et al., 2021b), mechanisms of self-regulation are implemented to prevent harm and exploitation of actors in the ecosystems (Cusumano et al., 2021). As the vulnerability of actors relying on personal services is even more menacing than on EPs like entertainment or ecommerce platforms, personal engagement platforms (PEPs), i.e. EPs implemented in the personal service sector which is traditionally reliant on physical, interpersonal interaction (Bitner et al., 2000), face additional challenges in gaining actor trust and engagement rely on more rigid means of self-regulation. We refer to self-regulation as non-governmental activities of companies or industry organizations which supplement governmental rules and guidelines. Consequently, self-regulation may not rely on forms of intervention such as legislation and penalties (e.g. taxes, subsidies, permits or licenses). Instead individual firms or industry organizations employ their respective regulatory measures or quality standards (Cusumano et al., 2021; Maitland, 1985).

To understand the need for (self-)regulation and a key reason for negative effects caused by the success of EPs, we need to consider the fundamental change of business models of EPs in recent years. As digital business models shift from exchanging goods and services to ones that monetize interactions, e.g., data- or advertising-based revenue models, EPs are instrumentalized to attract increasingly intense engagement of actors to grow market shares and reduce time spent on competing EPs (Kübler et al., 2021). Through this shift, beneficiaries of services and payers are different stakeholders that an EP must attract to build a proper monetization model. As a result, most business models do not necessarily rely on making the service’ beneficiaries lifes’ better but on keeping them engaged to monetize data and interactions with paying parties. This fueled fierce competition for attention and engagement among EPs building heavily on the satisfaction of short-term needs instead of long-term welfare, thus potentially nudging individual choices of actors against their long-term well-being (Osterle, 2020). This abundance in hedonically attractive services results in negative implications for social welfare such as technological addiction (Savcı & Aysan, 2017).

National, as well as, supranational regulation and self-imposed rules reduce market dominance and negative effects on welfare, including fake news, discrimination, depression, unhealthy behavior, etc. But, these regulations come with a high degree of complexity and risk to dampen global competitiveness and potentially beneficial innovations (Clemons & Wilson, 2018). Further, regulatory intervention is often too slow or too general to provide adequate principles that prioritize a common good on sectoral platforms (Van Dijck, 2020). Consequently, sectoral governance mechanisms to enforce contextually appropriate policies and reduce (potential) harm include certification, penalties, and self-regulation implemented and enforced by actors other than governmental organizations (Elhai, 2020). As the attention of scholars in platform research is mostly focused on notoriously successful EPs (de Reuver et al., 2018), e.g. Airbnb, Apple, and Facebook (Fu et al., 2018), there is a lack of discussion on EPs that are not themself dominating their market or aspire to challenge the status quo via revolutionary technological solutions. For these EPs, the debate on effective governance is far less prevalent compared to constant discussions on the power of big tech, fake news, and their impact on society.

Among others, there are several advances of platform operators (POs), i.e., actors governing EPs, in healthcare, emergency response and sustainability, to employ EPs that increase individual and social welfare (e.g. Fromm et al., 2021; Omar et al., 2019; Seidel et al., 2018). However, the more central a service is to individual privacy or the public interest, the higher the risk for losing actors’ trust when intrusion or misconduct occur. Consequently, to avoid harm for actors engaged on PEPs, POs often implement self-regulatory mechanisms proactively. Our research’s focus on the personal service sector is motivated by several considerations. Firstly, the implementation of mechanisms and self-regulation is formed by actor-specific characteristics, as well as, institutional and organizational arrangements in the context, e.g. a specific sector, in which value co-creation occurs (Storbacka, 2019). Therefore, a specific sector has been selected for our analysis to allow for the comparability between the EPs. As actor engagement in the personal service sector revolves around more intimate parts of the users’ lives, EPs in this sector need to be especially aware of actor preferences, privacy concerns, and establishing trust (Lattemann et al., 2019).

To explore and illustrate the regulatory mechanisms POs use to maintain individual well-being and social welfare, we conduct 14 semi-structured expert interviews with EP operators and discuss implications for governance of PEPs. Our related research activities are guided by two exploratory research questions:

1. Which activities and mechanisms foster engagement on engagement platforms in the personal service sector?
2. What kind of governance mechanisms are implemented to prevent harm on engagement platforms in the personal service sector?

Before adequate regulatory mechanisms are in place, POs themselves may contribute to social welfare and individual well-being of users. This research provides insights on how POs drive engagement while limiting themselves beyond regulatory standards to attract and bind actors. By highlighting how certain industries adapt to a social consensus in their context, we contribute to the current discussion on EP governance, social welfare, and individual well-being. The personal service industry is a significant driver of employment and growth in developed countries such as the DACH region (Germany(D), Austria(A), Switzerland(CH)) (BMBF, 2016). However, despite recent advances in digitalization, this sector is among the least digitized and technologically disrupted markets (BMBF, 2016). An exploration of adequate design and governance mechanisms could disseminate practical insights on how to prevent harm and foster social welfare in a sector of high social importance. Thereby, we contribute to recent interdisciplinary advancements in information systems literature that highlight the need to review societal and individual consequences of digitalization on individual and social welfare (e.g. Clemons & Banattar, 2018; Clemons & Wilson, 2018; Osterle, 2020; Pohlmeyer, 2013). These discussions are driven by the need to “minimizing the harm caused by technology’s rapid disruption of society” (social welfare computing) (Clemons et al., 2021, p. 6641) and describing the design of environments to foster individual well-being (positive design) (Desmet & Pohlmeyer, 2013).

## Theoretical background

### Engagement and value co-creation

Traditional producer-consumer relationships are increasingly replaced by a relational perspective that recognizes the co-creative interaction of heterogeneous actors in complex ecosystems, thus, actor engagement becomes a central concept in marketing and experience design (Brodie et al., 2011**;** Vargo & Lusch, 2011**;** Vivek et al., 2012). The service logic provides a fitting theoretical lens to assess and foster engagement (reliant on value co-creation) on EPs that are part of a larger engagement ecosystem (Breidbach et al., 2014**;** Lohrenz et al., 2021a**;** Lusch & Nambisan, 2015**;** Schreieck et al., 2016). In line with the suggestion from Ojasalo and Ojasalo (2018), we refer to service logic as an umbrella term for concepts of the service-dominant logic (Vargo & Lusch, 2004), service logic (Grönroos, 2011), and customer-dominant logic (Heinonen et al., 2010) which are tightly interwoven and inherently focused on the concept of value co-creation. Engagement, i.e. “a psychological state that occurs by virtue of interactive, co-creative customer experiences with a focal agent/object in particular service relationship” (Brodie et al., 2011, p. 259), explains how actors, e.g. customers, POs, or service providers, establish an interactive relationship with a focal agent or object to create not only an instrumental but also an experiential value(**−**in-use) for themselves (Grönroos, 2008**;** Vargo & Lusch, 2008). By that, the concept of engagement highlights an iterative process that builds trust, commitment, and individual well-being of actors, based on satisfying service experiences (Bowden, 2009**;** Geiger et al., 2020). A value-in-interaction is derived by actors based on several aspects, i.e. convenient access to adequate services (matching), improving their well-being based on the provided output and experience (service), and establishing a valuable connection to one or multiple actors (relationship) (Geiger et al., 2020**;** Robra-Bissantz, 2021). Unfavorable interactions, i.e. value co-destruction, however, may harm the service experience and reduce trust, commitment, and individual well-being (Luo et al., 2019). Additionally, unfavorable outcomes may result from externalities of the value co-creation of others. E.g., Airbnb may provide authentic and convenient services to tourists at the expense of long-term residents (Clemons et al., 2021). Consequently, value co-creation should be fostered by a recombination of practices, processes and institutions to “serve a human purpose” (p. 15) (Akaka & Vargo, 2014) and consider consequences for the overarching ecosystem (Lusch & Nambisan, 2015).

Fostering social and individual welfare gained growing attention in recent years, as technological platforms and applications experience unprecedented growth and increasing actor engagement. E.g. positive design proposed that the primary aim should be to design technological tools, e.g. an EP, in such a way that users are intrinsically motivated to engage, by satisfying their needs and, ideally, their individual well-being is improved as well (Desmet & Pohlmeyer, 2013**;** Peters et al., 2018**;** Riva et al., 2012). Thereby, positive design is concerned with designing environments that enable and stimulate human flourishing, therefore, foster well-being (Desmet & Pohlmeyer, 2013). Objective well-being is the degree to which the external requirements for a high quality of life, e.g. nutrition, living environment, are met. Whereas subjective well-being represents a personal perception about the quality of their respective life (Krueger & Stone, 2014). Subjective well-being can be fostered by allowing the actors to be autonomous and competent and increasing their perceived relatedness (Peters et al., 2018), as proposed by the self-determination theory (Ryan & Deci, 2000).

Considering societal issues that emerged with the mostly unregulated growth of technological platforms, the discipline of social welfare computing (Clemons et al., 2021**;** Clemons & Banattar, 2018**;** Clemons & Wilson, 2018), focuses on identifying problems associated with the rapid disruption of society caused by technology and formulating adequate responses to minimize the resulting harm. As highly engaging platforms are at the center of the current discussion, analyzing and designing appropriate governance mechanisms to foster EPs and the externalities they create, is of growing importance.

In this context, the penetration of markets through platformitization has been abrupt compared to similar technological evolutions of the past (Clemons et al., 2021). As a result, incumbents, novel actors, researchers, the public, and policy makers have yet to evaluate the “do’s and don’ts” for society associated with ecosystems, platforms, and their operators. A social consensus about contextual and sectoral rules has not been reached. Thus, putting effective regulation in place to impose common standards for commercial and social aspects remains a challenge as policy makers struggle to find the right means of regulation (Clemons & Wilson, 2018**;** Schreieck et al., 2019). National and supranational frameworks regulate market concentration, freedom of information and speech, as well as, privacy rights, mostly to target big tech companies or basic human rights (Van Dijck, 2020**;** Wahyuningtyas, 2019). Diversified sectoral platforms, however, are operated by heterogeneous organizations, such as established organizations, startups, as well as, governmental and public actors. As a result of the rapid advancement of society and technology, they implement self-regulation to prevent negative effects on societal and individual welfare (Elhai, 2020**;** Van Dijck et al., 2018). While established governance mechanisms to foster engagement are needed for EPs to be (economically) successful, depending on the context, culture, and sector-specific institutions need to be implemented to prevent potential misconduct or negative externalities. The implementation of governance mechanisms and self-regulation is an iterative process that is reconfiguring, based on how value or welfare is derived, how value co-creation is enabled or fostered, and what self-regulation is in place.

### Engagement platforms (EPs)

In recent years, EPs have become an emerging topic in service and co-creation research (Breidbach & Brodie, 2017; Fischer et al., 2020). EPs act as mediators among actors in service ecosystems that improve the exchange, provision, and commercialization of resources and services (Bidar et al., 2016; Frow et al., 2015; Lusch & Nambisan, 2015). The success of EPs is therefore directly reliant on its ability to allow and improve value co-creation, i.e., the process in which diverse actors integrate resources for their mutual benefit, and service innovation, i.e., the improvement of service experience related to value co-creation processes (Lusch & Nambisan, 2015). Such service innovation may result in new or novel services and the incremental improvement of existing offerings.

To vitalize co-creation activities on EPs and build actor engagement, different ways of organizing users to reach innovation opportunities (structural flexibility), as well as, mechanisms to understand and foster user interactions in a network (structural integrity) need to be considered and designed (Lusch & Nambisan, 2015). This effort, however, requires close attention to how individual actors within the ecosystem are influenced by the ecosystem’s structural properties (Edvardsson et al., 2011). Consequently, the EP design should be based on co-creative service innovation involving heterogeneous actors (Robra-Bissantz & Lattemann, 2006). Realizing structural flexibility and structural integrity as competitive advantages requires a critical mass of actors on the EP (Tiwana, 2015). As EPs are usually home to two or more distinctive actor groups (e.g. suppliers and buyers), an initial and potentially persisting challenge of balancing and growing an actor base with complementary or rivaling interests exists (Gawer & Cusumano, 2014).

To identify and categorize design requirements regarding balancing, growing, and governing an actor base, we conducted an extensive literature review (Fischer et al., 2020) following the systematic literature review process proposed by Webster and Watson (2002). Relying on Scopus and Google Scholar (specifically employed for unpublished or grey literature) as databases, 1169 articles matching our iteratively defined search string (“service platform*” OR “digital platform*” AND “Design Guideline*” OR “User Experience” OR “Design Requirement*” OR “Design Factor*” OR “Design Principle*” OR “Design Method*”)” have been screened to review the existing literature four design categories were derived that provide an overview of essential success factors for EP: (1) easing the entry, (2) identifying mutual problems and needs, (3) supporting value co-creation and (4) facilitating service innovation (Fischer et al., 2020).

Easing the entry encompasses activities that support a continued influx of new actors, e.g. by lowering the barriers to adapt to existing processes and cultures (Göbel & Cronholm, 2016) and collaboratively developed pricing and cost mechanisms that remain fair throughout the existence of the ecosystem, thereby, ensuring a motivating environment for established and new actors on the EP (Blaschke et al., 2019). Yet, lowering the costs of entry may also result in low switching costs, thus, encouraging multi-homing or actors switching to other EPs (Hein et al., 2019).

As actor resources and opportunities for co-creation of value on the EP remain dynamic due to the ever-changing external and internal environment, identifying mutual problems and needs provides EPs with a more strategic and aligned direction. Effective and efficient resource allocation and mobilization to drive service innovation on the EP are improved by e.g. utilizing information technology to identify and initiate co-creation and service innovation opportunities or by involving parties not yet included in the service ecosystem of the EP (Blaschke et al., 2019; Göbel & Cronholm, 2016).

Supporting value co-creation is a pivotal property of an EP. Consequently, EPs are supposed to establish institutions, i.e. formal or informal rules, norms, and beliefs (North, 1991), that improve the exchange of services (Blaschke et al., 2019). Furthermore, considering institutional ties of social and economic actors on EPs informs a more holistic understanding of actor engagement (Breidbach et al., 2014). Identifying and influencing interrelated sets of institutional arrangements affords more predictable and meaningful social interactions (Vargo & Lusch, 2016).

Activities and mechanisms to improve co-creation include the involvement of external actors and communities (Blaschke et al., 2019; Heinonen et al., 2010), the coordination of interaction, and the provision of freedom to (collaboratively), as well as the introduction of new and improved value propositions among heterogeneous actors (Aulkemeier et al., 2019). While the support of value co-creation in the service ecosystems that utilize EPs is essential (Göbel & Cronholm, 2016), attracting and maintaining a critical mass of actors, relies on the ability to introduce new, as well as, to improve the existing value propositions, and the overall service experience (Breidbach & Maglio, 2016). Therefore, facilitating service innovation, i.e. the improvement of the service experience, e.g. by establishing shared innovation processes (Göbel & Cronholm, 2016) and providing co-design opportunities with customers and third parties is essential to the long-term success of EPs (Aulkemeier et al., 2019; Blaschke et al., 2019; Spagnoletti et al., 2015).

As illustrated in the subsections above, POs rely on mechanisms to foster engagement on their platform ecosystem. Yet facing hazards or tangible negative consequences for well-being, they employ self-regulatory measures to prevent social and individual harm on the EPs. Drawing from these concepts, the following sections elaborate our research approach to explore what activities and mechanisms are employed by POs to foster value co-creation while preventing individual and social harm on PEPs.

### Methodology

As governance mechanisms and self-regulation on PEPs are scarcely researched, the area can be considered a nascent field of theory (Edmondson & Mcmanus, 2007). To explore the phenomena in this research context, we conducted 14 expert interviews with POs of PEPs. To ensure that the interviewees are qualified, we interviewed either founders, CEOs, or managers of two-sided B2C EPs, in the personal service sector, that have existed for at least two years and evince maturity by employing structured processes to innovate their existing offerings (Parker et al., 2016). Thereby, we were able to ensure that the PEPs had to adjust their practices over time and adopted successful governance mechanisms to stay in business. The complexity of governance mechanisms and institutions surrounding them, motivated an exploratory, qualitative research design, employing semi-structured expert interviews to retrieve rich, detailed, and evocative data to inform our analysis (Bryman & Bell, 2007; Edmondson & Mcmanus, 2007).

For the selection of qualified candidates, a list of 136 relevant PEPs with active communities in the DACH region (Germany (D), Austria (A), and Switzerland (CH)) was composed based on publicly available data. We emailed the contacts and received 20 responses, Subsequently, we re-evaluated if they fit into the PEP category and have been in the market longer than two years, to then set up an interview appointment with 14 of them (Table 1). The interviews lasted on average 52 minutes and participants were on average 40 years old, with three female and eleven male experts. The interviews were conducted via phone and video conference tools.

**Table 1:** Interview partners

*Platform*	*Domain*	*Modes of interaction*	*Position*
Animus	Living Quarters	Virtual and physical (optional)	Bus. Dev. Mgr
Care	Childcare	Virtual and physical	New Bus. Mgr.
CraftNote	Craftsman Support	Virtual and physical	Founder
Dear-Employee	Job Health	Virtual and physical	CEO
Einkaufshelden	Local Shopping	Virtual and physical	Founder
ExtraSauber	Cleaning Services	Virtual and physical	Founder
DScreening	Job Health	Virtual and physical (optional)	Founder
Jobruf	Consumer Services C2C	Virtual and physical	Founder
Feelix	Finance and Insurance	Purely virtual	Sales Manager
MyHammer	Craftsman Services	Virtual and physical	CEO
MyHelpBuddy	Multi-lingual Assistance	Virtual and physical (optional)	Founder
Nebenan	Neighborhood Activities	Virtual and physical (optional)	Founder
Notfallmamas	Childcare	Virtual and physical	CEO
Yoopies	Care, childcare, cleaning, and teaching	Virtual and physical	Sales Manager

To conduct the semi-structured interviews, a guideline was developed based on existing design requirements and principles for digital service platforms (Fischer et al., 2020) to identify underlying activities and governance mechanisms. Semi-structured interviews were chosen to give the interviewees enough freedom to elaborate. The interview questions were aimed at enhancing or solidifying the identified categories of the literature analysis (Fischer et al., 2020). The interview guideline consisted of five parts: (1) introduction of the interviewee and basic information of the company, (2) elaboration of functional aspects and actor constellations on the platform, (3) elaboration of interaction and value co-creation activities, (4) elaboration of service innovation processes, approaches and actor engagement and (5) elaboration of governance features.

The interviews were conducted between May 7th and September 23rd, 2020. While the implications of COVID-19 already affected the business of the PEPs in this period, further research would be necessary to understand long-term effects of the pandemic on PEPs. The interviews were transcribed and coded in MAXQDA 2020. Before the first expert interview, an initial list of deductive codes consisted of the four categories derived from the literature analysis, i.e. “easing the entry”, “mutual problems and needs”, “co-creation” and “service innovation” (Fischer et al., 2020). Since several coding cycles are needed for analyzing qualitative data (Saldaña, 2013), the coding was conducted in three cycles, going from general labeling to categorization. The coding was done independently by the authors and afterwards discussed collaboratively to improve the robustness of our findings based on consensus agreement. In the first coding cycle, we used the four deductive codes as labels. After becoming familiar with the data, we established more specific codes and subcodes in the second inductive coding cycle. We opted for inductive coding as it allowed us to better reflect the statements of experts without being restricted only by theoretical literature (Linneberg & Korsgaard, 2019). For example, “Facilitating Service Innovation” got subdivided into subcodes like “Facilitation of Co-Design Activities” or “Improved Discovery of Innovation Opportunities”. Thereby, in addition to the initial set of labels, 34 codes were created inductively with 382 codings, in total. These codings included governance mechanisms and self-regulations mentioned by the POs and were later analyzed and grouped in workshop sessions. In general, a mechanism describes activities, functions or other means that are employed to reach particular aims (Gregor et al., 2020). In the evaluation of the 14 interviews, saturation was reached after 12 interviews as no additional information and codes were found. In the third and last cycle of coding, the specific codes and subcodes of the second cycle were grouped in the four design categories derived from the literature analysis and analyzed with respect to self-regulation, individual well-being and social welfare. Thus, in the results section, our findings are presented accordingly.

### Empirical setting

All EPs in our sample are offering personal services. Hence, these PEPs serve as a diversified set of EPs for the exploration of this sector. The comparison of the analyzed PEPs based on three criteria highlighted illustrates how different domains a) vary in the amount of information and choice they present their users with (cf. Clemons et al., 2019), b) implemented more self-regulatory mechanisms to prevent harm and build trust with their users, and c) rely on a higher degree of interaction in the real-world than others. While Feelix is an app-based platform that relies purely on virtual interaction with partners and users, peer-to-peer/business-to-consumer-PEPs such as Craftnote, Extrasauber, MyHammer, Jobruf, and MyHelpBuddy offer services, were actors partly or to a high degree require entrance to the home or living environment of each other. Here, the information related to the service providers provides the highest level of detail in the sample, including detailed descriptions, ratings, and histories. Einkaufshelden, Nebenan, and Animus build on regional communities that most likely will interact in person but may also limit their interaction to the virtual realm. The information of actors is limited to basic information. DScreening and DearEmployee offer individual services related to health management. While questionnaires may be purely virtual, other services, e.g., vaccination or consultation, need in-person contact outside the actors living environment. Available information on service providers is highly detailed and success managers will assist with finding the right service. Childcare services like Care, Notfallmamas, and Yoopies necessitate human interaction with children and implement rigorous measures to certify and verify good conduct and proficiency of caretakers and other actors.

PEPs with high levels of physicality and proximity to the living environment of actors, either differentiate and build trust by allowing offerers to provide robust information about themselves or provide limited information publicly while ensuring good conduct through verifying identification or certifications, personal onboarding, and training. Conversely, PEPs with a low level of physicality, e.g. Feelix, or touch points less related to the personal space of actors, e.g. Einkaufshelden, reduce barriers of entry and identification. In line with Clemons et al. (2019), we assume that these differences are deliberate and part of the EPs strategies, e.g., consciously applying an efficiency strategy if appropriate.

## Results

Based on the four essential categories for EPs, (1) easing the entry, (2) identifying mutual problems and needs, (3) supporting value co-creation, and (4) facilitating service innovation, we will present governance mechanisms employed by the POs. Additionally, we highlight the contextual or sectoral characteristics if needed to understand nuances in the context of personal services. To increase clarity, these inputs coming directly from the experts are highlighted with quotation marks. The mechanisms to foster engagement, the respective self-regulatory measures applied by the PEPs, as well as, intended contributions to individual and social welfare are summarized in Table 2 according to the order of appearance. The overarching mechanisms to foster engagement are written in italic print and enumerated *M1-M8* according to their appearance in Table 2. M1-M8 are also complemented by self-regulatory mechanisms to prevent harm or misconduct on the platform. By that POs intend to improve welfare of actors in the PEPs’ ecosystems.

**Table 2:** Summary of governance mechanisms and intended effect on individual well-being and social welfare

Mechanism to foster engagement	Self-regulatory mechanism to prevent harm or misconduct	Intended outcomes for individual well-being and social welfare	Related categories
Instrumentalize existing platforms to attract a constant influx of actors (M1)	Human moderation of content, verification of actors, and extensive support and training. Avoidance of aggressive marketing that might be perceived as predatory behavior.	Ensure positive interactions and build trust to allow meaningful relatedness to increase well-being and social connectedness (e.g., on neighborhood PEPs).	Easing the entry Supporting value co-creation
Create transparency about quality standards, rules, and rating (M2)	Employ and enforce rulesets to prevent misdemeanor, misconduct and create binding standards for communication and service quality.		Easing the entry Supporting value co-creation
Implement fair risk-based pricing and cost mechanisms collaboratively (M3)	Collaborative development and adjustment of cost and liability mechanisms with focal actors.	Enable actors to engage in value co-creation to foster their well-being, without leveraging power as central PEP. Ensure the implementation of fair payment for service providers by contract, to avoid exploitation and unregistered work.	Easing the entry Identifying mutual problems and needs
Prevent harm through certification and verification (M4)	Certification of relevant aspects to prevent harm, e.g., testing the safety of the payment system or privacy, verification of real neighbors, verification of qualification, and good conduct in care and household-related services.	Prevention of misconduct affecting the personal space or safety of actors as well as black market activities.	Easing the entry Identifying mutual problems and needs Supporting value co-creation
Consider virtual and physical touchpoints (M5)	Events and physical material, such as books, welcome packages, etc., provide rich symbols and afford effective communication of institutions to actors and scouting for new trends and issues.	Identification of overarching issues by direct interaction with actors and implement adequate rules to address negative externalities, e.g., uninsured, or underpaid work, to contribute to social welfare.	Easing the entry Identifying mutual problems and needs Supporting value co-creation
Provide opportunities for feedback(M6)	Offer adequate support, e.g., a 24/7 hotline in emergency childcare, and opportunities to report feedback or misbehavior.	Appropriate means of support and intervention should account for events that need swift reaction to prevent harm to individuals or the PEPs ecosystem.	Facilitating service Innovation
Reduce tracking and advertisement (M7)	Advertisement, selling user information, and extensive tracking accompanied the success of EPs, yet in the personal service sector, POs opted to reduce tracking and data collection as monetizing context, such as childcare, to increase trust.	Sovereignty of personal data affords actors on PEPs with control and comfort related to data security.	Easing of entry Identifying mutual problems and needs
Design for usability (M8)	Use common standards and transparency to avoid misleading users.	Barrier-free design affords the inclusion of (eligible) users. Eligibility refers to valid access to services not savviness.	Easing ease of entry Facilitation service innovation

### Easing the entry

An EP needs a critical mass of users to provide support and sustain value co-creation activities and create a competitive advantage that attracts additional actors (network effects). To foster a steady influx of new actors, who provide and demand offerings, POs employ various mechanisms to attract and bind actors to foster resource integration. As a result, POs need to increase the visibility of the PEP among potential actors. Therefore, POs *instrumentalize existing platforms to attract a constant influx of actors (M1)*. These platforms may include e.g., app stores, social media platforms, physical events, marketing campaigns, and B2B partners. While this comes as no surprise, the initial service experience of actors, especially in the PEP, needs to be deliberately crafted to the expectations of users and relies on establishing a near-immediate trustworthy relationship with the actor. “So everything that’s in this app was based on the requests of daycare centers at some point. And that’s how we handle it now.”

To further increase the chance for long-term engagement with actors, POs *implement fair risk-based pricing and cost mechanisms collaboratively (M3)* with actors, to establish mutual trust and lasting relationships. This self-regulatory measure can positively influence well-being through actors’ optional participation in decisions and the provided transparency. Also, all POs *foster trust through certification and verification (M4).* They also transparently employ sets of rules that avoid misconduct and provide binding quality standards, by defining what the services include or exclude. Regarding social welfare, e.g., this can lead to a prevention of exploitation of labour or undeclared work, as confirmed by Yoopies and ExtraSauber.

Except for Feelix, PEPs deliberately c*onsider virtual and physical touchpoints (M5).* POs, e.g., Care, Nebenan, and Animus, also provide physical welcome packages, books with exceptional examples of actors/offerings, or complementary commercial material for actors to build a stronger relationship, acknowledging the physical aspects of a PEP ecosystem that often involve a high degree of real-world interpersonal interaction in the actors living environment.

Providing such support ensures that new actors are introduced to the PEP’s rules, processes, and shared worldviews to *create transparency about quality standards, rules, and ratings (M2)*. By initially supporting and enforcing how interaction among actors is supposed to be, POs provide new actors with a shared fundamental understanding, establishing a consensus. This shared understanding facilitates the strengthening of relatedness to the PEP, which is reflected in users’ engagement and well-being. E.g., Einkaufshelden, ExtraSauber, and Care deliberately discuss and communicate their understanding of the EP with new and central actors in face-to-face settings. “We deliberately don’t do that via any tutorial videos; we always do it in person.”

An explicitly formulated and shared understanding also lowers the necessary costs and efforts of new actors that join EPs. This starts with many activities related to features and practices *designed for usability (M8)* and accessibility, based on both virtual and real-world components. Virtual components are developed with a strong emphasis on easy-to-use interfaces, the availability of common payment methods, FAQs, video tutorials, and other helpful resources, thereby reducing initial reluctance factors. Real-world components include training, regular visits, the attendance of actor conferences and socializing events, etc. Designing PEPs that are easy to use and barrier-free, that are especially necessary for neighborhood PEPs, enables users to interact with the PEP transparently and also promotes social welfare, for instance regarding the goal of inclusion of everyone.

The final hurdle to attract and bind actors to PEPs is related to trust and the prevention of misconduct. Therefore, PEPs offering childcare and household services cannot only depend on self-regulation to ensure the well-being of e.g. minors. Here POs stated that they rely on thorough verification of new applicants to prevent the spread of unwanted advertisement, fraud, and other abusive behavior that, in this context, are connected to extreme risks, and check legal documents such as IDs, business licenses, and criminal records, and conduct interviews as well as verification calls.

To further increase the trust in their practices, some POs, like e.g., Care, *reduce tracking and advertisement (M7)* and restrict themselves only to tracking fundamental metrics that greatly limit their analysis of on-platform activities, strongly signaling that they do not track and monetize user data via their childcare platform.

### Identifying mutual problems and needs

In order to solidify the overall competitive position of an EP in ever-changing markets, mutual problems and needs of actors require a constant assessment to address internal and external stimuli. The collaborative identification of these factors decreases misguided resource allocation and innovation activities on the EP while increasing the transparency and awareness of latent capabilities among actors, as well as a common understanding and direction for future developments. This also allows POs to *implement fair risk-based pricing and cost mechanisms collaboratively (M3).*

Most EPs in our interview sample do not orchestrate strategic initiatives to sense and address mutual problems and needs collaboratively. This is especially evident for IT-based sensing of needs and problems that across all EPs is limited to the analysis of clickstream data exclusively considered for user experience optimization and activity reports. While optimization and analyzing trends are undeniably important, these IT-supported activities are limited in observing the big picture. PEPs with business models that cater to childcare or similar sectors (e.g., Care, Yoopies), *reduce tracking and advertisement (M7)* (e.g., tracking click-stream data). In this aspect, they regulate themselves, so as not to lose the trust of parents by tracking or even monetizing any data associated with information that may relate to children. In this regard, for instance, they heavily restrict themselves in the selection of hosting services from other countries even if this hinders the implementation of new functionalities.” If at some point you find a tool, which is totally privacy compliant, used in Germany, and so on. I’ll take it right away.”

POs actively engage key actors to observe and implement new initiatives. By giving actors a choice whether they want to participate, but at the same time strengthening the connection with the PEP by including the actors, their well-being is raised. This, of course, also leads to a stronger willingness of actors to continue engaging with the PEP. One PO states: “Ultimately, it is the person behind it who should use the application and find the digital platform useful.”

By attending and hosting networking events, EPs *consider virtual and physical touchpoints (M5)* alike to receive first-hand feedback, so that problems and market changes can be identified early on. This enabled e.g., both Yoopies and ExtraSauber to identify that there is a large societal interest to decrease undeclared work in the household service sector. Therefore, they *foster trust through certification and verification (M4)*. By addressing this social welfare issue, they secured non-monetary support from the government and attracted companies and institutions affected by this.

With trends, problems, and needs of heterogeneous actors quickly appearing and changing, all POs adopted agile development approaches to implement new features quickly. This can be especially helpful, if new regulations, such as the General Data Protection Regulation, need to be implemented.

### Supporting value co-creation

An instance of value co-creation of actors will most likely evolve to engagement if they have a positive service experience. Therefore, fostering actor engagement relies on the PEPs’ ability to identify the heterogeneous needs, as well as their relationships and resource integration processes. POs, consequently, implemented several mechanisms and co-design processes with other actors to appeal to their respective preferences and to ensure critical needs are accounted for. To enhance the efficiency and effectiveness of value co-creation on the PEPs, a primary activity is to facilitate access to appropriate resources. PEPs, therefore, try to create fast-growing actor bases to offer access to optimal resource configurations, which fulfill the specific needs of the actors. Both EPs and PEPs rely on growing an actor base that is balanced so that actors of two- or multi-sided markets are provided with attractive opportunities to co-create value, build engagement, and derive a benefit for them or others. Therefore, POs increase the total number of active actors by *instrumentalizing existing platforms to attract a constant influx of actors (M1)* and foster engagement to the PEP via social features. To compensate for an initially small community, PEPs, e.g., Animus and MyHelpBuddy, actively engage service providers and other actor groups in areas where users encounter a lack of offerings, connect them to prevent dissatisfaction in early stages, and build local or specialized communities manually. POs, e.g., MyHammer and Nebenan, also share stories via social media to build trust, inspire new interaction opportunities, and attract new users. By that, they aim to increase the engagement of actors on the PEP. Conversely, aggressive online marketing via social media may also endanger the EP’s attractiveness and credibility, thus having limited effects on actor growth and engagement. Instead, most POs adopted a personal touch to their communication and testimonials, as a central part of their social media campaigns.

While attracting and connecting actors of a PEP via various touch points is a key driver to afford growing and improving access to needed resources, several POs deliberately restrict the access to their PEPs. As good service experiences rely on offerers meeting the expectations of beneficiaries, most PEPs in our sample *create transparency about quality standards, rules, and ratings (M2)* for value co-creative activities, e.g., interaction among actors, quality of services, qualification. On PEPs with social features, POs, e.g., Nebenan, rely primarily on netiquette and trust the actors to follow these rules voluntarily. Thus, allowing for self-regulation among the actors themselves. We often found that, e.g., in neighborhood PEPs, the actors govern interaction through their own social rules. Via external social platforms or social features on the PEPs, individual well-being may be fostered through interpersonal relationships with a community or through finding highly personalized services and improvements to personal life situations. While several formal and informal rules have been introduced to fill a lack of legal policies and effective enforcement, additional self-regulatory norms emerge and change iteratively in the actor ecosystems. These rules are based on a sectoral and cultural consensus that may heavily vary across sectors and regions. POs, usually focal actors with competence and authority, closely view interaction and update mechanisms according to these changes.

Even though EPs are a major driver to enhance actor engagement, the interaction afforded by PEPs is inherently reliant on both virtual and real-world touchpoints. Therefore, regional presence plays a larger role compared to EPs in other sectors, thus deliberately *considering both virtual and physical touchpoints (M5)*. One interviewee stated that: “this is what guides us, so we say we don’t want to be an online tool that draws people online for its own sake, but rather a platform that is a useful online tool and always results in real added value offline as well.”

Value co-creation and engagement are the pivotal elements of PEP design and success. Through the integration of their resources, actors create benefits for themselves and others. Especially in the personal service sector, these benefits are tied to social welfare, e.g., in care and education, and individual well-being, e.g., improving individual living conditions through self or household-related service. Childcare PEPs perform services that heavily affect large portions of children’s lives and influence their education and socialization. Household-related PEPs afford cleaners and other service providers with access to homes. Compared to regular EPs of other sectors, there are rigorous self-regulations implemented to *foster trust through certification and verification (M4)* and foster well-being and social welfare. To prevent misconduct, e.g., data theft, theft, discrimination, harassment, fraud, or undeclared work, POs additionally verify legal documents of actors depending on the context: Personal IDs, certificates of good conduct, training certificates (childcare), home address (neighborhood platforms), business registration (household-related services), etc., to ensure that actors are competent and trustworthy. Thereby, they are filing a legal void which does not affect EPs in different sectors similarly.

### Facilitating service innovation

As markets are continually changing and actor demands increase, EPs must ensure feasible and sustainable service innovation to maintain continuous actor engagement and prevent harm, resulting from adopting rapidly evolving trends of digitalization without adequate mechanisms to ensure their positive utilization. While the interviewed companies principally claim that service innovations are driven by the users, these are also dependent on monetary resources and development capacities. Regardless, there are functionalities that must be developed due to e.g., data protection issues and do not necessarily ensure the affected actors’ approval, as general regulation and mechanisms to avoid misconduct, must be implemented swiftly.

To not solely rely on internal and actor input to *consider innovation of virtual and physical touchpoints (M5)*, e.g., Animus, actively scouts for new trends by participating in trade fairs and informing themselves through current literature. To further facilitate the discovery of needs and new opportunities, the POs in our sample frequently mentioned that they *provide opportunities for feedback (M6)* and treat all actors on eye level to create an open feedback culture. Candid communication allows for ideas and potential sources of harm to be addressed and discussed openly. Opportunities to provide user feedback, e.g., through technical implementation, should be created and actively demanded from all actors. This also includes feedback about PEPs, e.g., through bug reports to *design for usability (M8)*.

Some POs, e.g., MyHammer, have their own R&D departments, while others, e.g. Feelix, host open exchange rounds among their employees, where ideas can be shared. To manage this influx of ideas POs, e.g., Craftnote and Nebenan, rely on agile development methods to increase the speed of implementation and user-centeredness of the user-generated ideas. As the ideas and wishes of actors sometimes need further investigation, PEPs, e.g., Care and Animus, conduct user workshops and UX-tests to define novel solutions that surpass the original input and reinvent processes to foster actor engagement. This user proximity, in turn, promotes trust and ensures that concerns can be addressed promptly. Widely established features, such as rating or endorsement systems, help actors make informed decisions and allow actors to rate each other. Such transparent feedback mechanisms further solidify the implementation and application of formal and informal rules to value co-creation.

Further, to ensure high-quality services POs, e.g., Einkaufshelden and Yoopies, evaluate samples and accompany actors in the onboarding process. Additionally, rating systems are implemented that allow actors to rate each other. As a better rating is linked to higher visibility and trust of potential users, offerers and beneficiaries try to rank highly in the rating system. As breaking rules in actor interaction would lead to negative ratings, formal and informal institutions of the ecosystem are enforced, and actors have additional incentives to improve the service experience. As a result, the inclusion of these and similar governance mechanisms fosters a trust relationship with the PEP and the associated services. Building confidence in the PEPs increases continuous actor engagement, as actors expect additional positive experiences. As EPs compete for their users, new features and technologies are introduced at a fast pace. In the personal service context, on the other hand, PEPs may deliberately delay the introduction of novel features until self-regulatory measures are implemented, to avoid misconduct or harm that may disrupt a service experience, closely tied to one’s personal life.

PEPs are sometimes created as direct responses to societal issues. To name a few, MyHelpBuddy created a PEP to allow refugees to find translators and assistance with everyday challenges in a foreign place, Notfallmamas introduced a video-childcare-service for parents and children stuck at home due to the COVID-19 lock-down, and Yoopies and ExtraSauber work closely together with governmental services to reduce unregistered and uninsured workers in the household- and personal service sector. While other sectoral contexts address social welfare-related issues of similar scope, currently the personal service sector is among the industries with the lowest level of digitalization. Implementation and self-regulation of technological solutions, such as PEPs thus pose significant challenges to the POs in our sample and increases the need to find and adopt a social consensus on how to digitize services with high degrees of personal interaction and direct involvement in personal lives.

## Discussion

In this contribution, we provide new insights into PEP activities for practitioners looking for descriptive information, and for scholars of the service science and the platform literature, on how to enable and foster interaction on PEPs. Here we show that the essential issues are: not to compromise user trust through excessive data use, proactively implement new rules on user wellbeing before legislative changes make them necessary, and consciously limit growth acknowledging possible negative implications on social welfare. Thereby, we contribute to existing research gaps concerning a) applicable research that draws from service logic (Vargo & Lusch, 2017), b) the call for actionable information for practitioners designing EPs (Blaschke et al., 2019), and c) self-regulation in the personal service industry and their implications for individual well-being and social welfare. Our scholarly and practical contributions will be discussed in three sections: “avoid user distrust through excessive data use”, “proactively implement new rules for user well-being”, and “consciously limit growth”. While our research is descriptive in nature, the discussion section aims to provide practical insights to address present issues in platform design.

### Avoid user distrust through excessive data use

Since the availability and successful matching of resources on the EP are central to its success (de Reuver et al., 2018), initially, the EP has to earn the trust of actors through activities prior to the actual co-creation of value (Oh & Moon, 2016). In our sample, trust is often established at an early stage through personal contact among PO and users and can, later on, be supported or fully replaced by testimonials or reviews, after an appropriate number of actors has been reached. Additionally, the interviewed companies rely on self-imposed rules and request user authentication and, in some cases, even demand personal identity cards or business register entries, to prevent harm in an ecosystem. However, this established user’s trust must not be exploited to expand the POs own market power, as this may result in the tragedy of the commons, meaning the depletion of the resource where the original prosperity stems from (Cusumano et al., 2021). According to Horvitz and Mulligan (2015), the governance of data should be “use-based” to moderate the risks of so-called “category hopping,” in which characteristics or conditions are revealed that individuals might otherwise prefer to keep secret.

As PEPs in e.g. the childcare domain have demonstrated, practitioners should consider that excessive use of data may be considered appropriate in some domains but pose a substantial risk to individual well-being and safety in others. Ethical risks stemming from data misuse are widely addressed in literature. Here, discussions about privacy concerns regarding data use, include whether data should be processed client-side or server-side (Sinche et al., 2017), how to minimize data collection and storage (Tollmar et al., 2012), and what alternatives can be used, for example instead of video surveillance, to achieve greater trust among users (Garcia-Ceja et al., 2016).

### Proactively implement new rules for user well-being

Burr et al. (2020), raise the question, if ethical guidelines alone can be sufficient against the misuse of digital footprints or stricter legal frameworks are required. Our contribution to literature includes empirical evidence, that PEPs in particular resort to forms of self-regulation, as they are more susceptible to harm and misconduct due to personal proximity, in comparison to other EPs. This is in line with Elhai (2020), who argues that in areas where complex aspects require regulation, non-governmental organizations and trade groups intervene to offer certification to enforce common standards. This is especially the case when one or multiple sides of the market are growing fast and are expected to diminish the average service quality on the PEP, as they are also more prone to errors. Veisdal (2020) also reports that a vast selection of e.g. suppliers might lead to higher searching costs of actors.

Due to the rapid growth of EPs, the effects of digitization on the environment and society cannot only be considered in retrospect, as was the case in e.g., the agriculture industry. Also, many currently valid regulations cannot be applied to the digital economy (Clemons et al., 2021). Policies and rules must therefore be developed by proactively to ensure social welfare and prevent harm (Clemons & Banattar, 2018; Cusumano et al., 2021). The identified governance mechanisms can be used by practitioners to address pressing issues revolving around social welfare computing (Clemons et al., 2021) and to advance positive design to create EPs for user well-being (Desmet & Pohlmeyer, 2013; Lohrenz et al., 2021b; Peters et al., 2018; Yaden et al., 2018). Of these, social welfare computing mostly concerns activities that benefit the greater common good and may therefore require stricter regulation (e.g., General Data Protection Regulation) or stricter self-regulation, e.g., controlling legal documents that are meant to protect private individuals. Floridi et al. (2018) argue that compliance with the law is necessary, but insufficient, as there is more that can and should be done to protect the user. Similarly, it is not beneficial to wait with self-regulation until government agencies impose standards or rules, thus jeopardizing potential growth and further development of platform business (Cammaerts & Mansell, 2020).

The introduction of similar governance mechanisms is dependent on cultural, sectoral, and actor specific characteristics but should be ultimately guided by considerations of how to “improve people’s quality of life” (Osterle, 2020) or minimize harm resulting from technological disruption of society (Clemons et al., 2021). Ultimately, respecting the individual well-being and social welfare of users should always be at the forefront of value co-creation and may also provide an avenue of inspiration for EPs in other sectors.

### Consciously limit growth

As Clemons and Wilson (2018) argue, education about the social impact of big companies and their market dominance and data wealth should be addressed much earlier on, for example in higher education, to create a mindset shift among tomorrow’s leaders. Spiekermann et al. (2022, p. 250) say: “True value creation is not a matter of technology design alone but also strategy, corporate culture, and companies’ willingness to forgo some profit for the sake of community, integrity, and accountability.”

If practitioners manage not only to see their individual profit through the EP but also to consider the social welfare and the well-being of each individual, it may be possible to adequately substitute extensive regulation in sectors with specific institutions. Therefore, POs may consider positive design as guidelines (Peters et al., 2018) to enable autonomous choices, allow users to act competently, and increase users’ perceived relatedness on EPs (Lohrenz et al., 2021b). Against this backdrop, we contribute by offering empirical research on well-being and social welfare to inform adjustments or implementation of mechanisms regarding stakeholder management, business ethics, creating shared value, conscious capitalism, or other concepts related to corporate social responsibility (Carroll, 2016). Vice versa, the inclusion of frameworks from different disciplines (Clemons et al., 2021), such as Carroll’s pyramid of corporate social responsibility (Carroll, 1991), could provide an excellent basis for creating design theories (cf. Hevner & Chatterjee, 2010) related to EP design, governance and regulation.

### Future research and limitations

The activities and mechanisms presented can be implemented by POs to design existing or new platforms with regard to social welfare and personal well-being, and increase essential actor engagement. However, these governance mechanisms currently represent only examples in which a particular activity can be implemented and should be elaborated in further studies. E.g, Steur and Seiter (2021) have looked at how over 100 feedback mechanisms are implemented and summarized them in a complete design catalogue. Such an approach can be used for many of the mechanisms illustrated here. As governance mechanisms, and self-regulation, in particular, need to consider cultural, sector, and actor-specific characteristics (Elhai, 2020) our analysis of the personal services sector may only inform core activities and mechanisms in other sectors with great caution.

EPs and PEPs could differ, especially due to physical proximity, which might have changed during the Covid 19 pandemic (Otterbring, 2022). POs in our sample often rely on a mixed virtual and real-world approach regarding the onboarding and co-creation process. This physical component is reflected fittingly in the EP perspective that assumes physical touchpoints as integral factors of interaction between actors (Breidbach et al., 2014). Additionally, except for Feelix, all PEPs in our sample rely on or implied heavily that physical touchpoints are central to their value co-creation and interactions.

We briefly outline the heterogeneity of our sample in the methodology part but encourage, as well as, plan to conduct further analysis of specific domains to identify differentiation strategies of PEPs (e.g., based on the degrees of physical interaction, self-regulation, and robustness of information) or other EPs, as suggested by Clemons et al. (2019) for sharing economy platforms.

While the categorization and associated mechanisms to foster engagement on platforms are based on an extensive literature review (Fischer et al., 2020), the qualitative exploration of PEPs and sector-specific characteristics needs to be extended with further empirical research to contrast sectoral differences. Also, as the interviews have been conducted with experts in the DACH region, further research may compare the effect of regional and cultural differences with our results. Especially in the area of regulations and culture, European markets can differ greatly from, e.g., American or Chinese markets. Also, resulting from the deliberate choice to interview experts in the B2C sector we refrained from interviewing consumers, as the selection of experts for our interviews was more robustly ensured by relying on established and measurable criteria, such as job position and time in the market. Further, future research should extend this study by considering a multi-actor perspective that actively seeks to balance the interests of different actors and/or identifying similarities and differences to other sectors. Explorative and illustrative case studies guided by challenges related to individual and societal welfare should be extended e.g., to big tech platforms to illuminate current good and bad practices that exist in regulatory loopholes, or examine the use of mechanisms across start up, grow up, and maturity of platforms. Additionally, the effect of regional regulation or EP self-regulation (e.g., the ban of beauty filters on beauty advertisements in the UK or cosmetic surgery filters on Instagram) on other competitors and regional legislation should be examined to evaluate dynamics of legislation and self-regulation on competing EPs. Consequently, the mechanisms presented are not exhaustive and need to be explored further in future studies. Other mechanisms, e.g., of governmental and institutional theory, can be analyzed to gain a more complete set of mechanisms at the global level as well.

## Conclusion

EPs are increasingly popular and powerful means to enable co-creation and service innovation. Therefore, the optimal design of EPs is receiving growing attention in research and practice. In this study, we conducted expert interviews with founders, CEOs, and managers of 14 PEP companies to gain insights into essential success factors that could aid in designing better EPs. In addition, we identified and elaborated governance mechanisms concerning individual well-being and social welfare to identify activities for the increase of actor engagement. To analyze the interviews, we employed deductive and inductive coding, and were able to solidify the validity of four categories for EP design that we derived in a preceding literature review: (1) easing the entry, (2) identifying mutual problems and needs, (3) supporting value co-creation, and (4) facilitating service innovation. Further, we found that POs of PEPs employ mechanisms to foster value co-creation while balancing growth against risks of social and individual harm through self-regulation, as they are more vulnerable to misconduct due to personal proximity compared to other EPs.

Eight mechanisms and related self-regulatory measures have been identified on PEPs. These insights help platform operators to implement or adjust mechanisms that build more trust with actors and foster lasting engagement in accordance with economic, legal, ethical, or even philanthropic responsibilities. Based on these empirical insights scholars and practitioners may derive design principles and redefine their activities to build and foster interaction on future or existent PEPs, thus increasing long-term actor engagement as well as individual and social welfare.

While the POs of PEPs deliberately employ self-regulatory measures to prevent harm, other sectors are employing mechanisms to rapidly grow or dominate their market segment despite negative consequences. Our exploratory research provides a starting point to inform the growing field of welfare computing on how to design PEPs to reduce negative externalities in the personal service context. This research can be expanded to other sectoral and cultural settings, evaluating a) similarities and differences, b) the effect of self-regulatory measures on PEPs, and c) how to implement mechanisms and self-regulation effectively. By that, limitations of this research, such as cultural and sectoral focus on the personal service sector in the DACH region and a lacking evaluation of effects on societal and individual welfare are addressed. In this regard, with the inclusion of various actor groups, specifically users, the findings of this research may be advanced to foster comparability and robustness.
